# Mechanistic modeling of sulfur-deprived photosynthesis and hydrogen production in suspensions of *Chlamydomonas reinhardtii*

**DOI:** 10.1002/bit.25023

**Published:** 2013-09-11

**Authors:** C R Williams, MA Bees

**Affiliations:** 1 British Antarctic Survey, Natural Environment Research CouncilHigh Cross, Madingley Road, Cambridge, CB3 0ET, UK; 2 Department of Mathematics, University of YorkYork, YO10 5DD, UK

**Keywords:** hydrogen production, sulfur deprivation, photosynthetic growth, light limitation, mechanistic model, *Chlamydomonas reinhardtii*

## Abstract

The ability of unicellular green algal species such as *Chlamydomonas reinhardtii* to produce hydrogen gas via iron-hydrogenase is well known. However, the oxygen-sensitive hydrogenase is closely linked to the photosynthetic chain in such a way that hydrogen and oxygen production need to be separated temporally for sustained photo-production. Under illumination, sulfur-deprivation has been shown to accommodate the production of hydrogen gas by partially-deactivating O_2_ evolution activity, leading to anaerobiosis in a sealed culture. As these facets are coupled, and the system complex, mathematical approaches potentially are of significant value since they may reveal improved or even optimal schemes for maximizing hydrogen production. Here, a mechanistic model of the system is constructed from consideration of the essential pathways and processes. The role of sulfur in photosynthesis (via PSII) and the storage and catabolism of endogenous substrate, and thus growth and decay of culture density, are explicitly modeled in order to describe and explore the complex interactions that lead to H_2_ production during sulfur-deprivation. As far as possible, functional forms and parameter values are determined or estimated from experimental data. The model is compared with published experimental studies and, encouragingly, qualitative agreement for trends in hydrogen yield and initiation time are found. It is then employed to probe optimal external sulfur and illumination conditions for hydrogen production, which are found to differ depending on whether a maximum yield of gas or initial production rate is required. The model constitutes a powerful theoretical tool for investigating novel sulfur cycling regimes that may ultimately be used to improve the commercial viability of hydrogen gas production from microorganisms. Biotechnol. Bioeng. 2014;111: 320–335. © 2013 The Authors. Biotechnology and Bioengineering Published by Wiley Periodicals, Inc.

## Introduction

**Figure 1:**
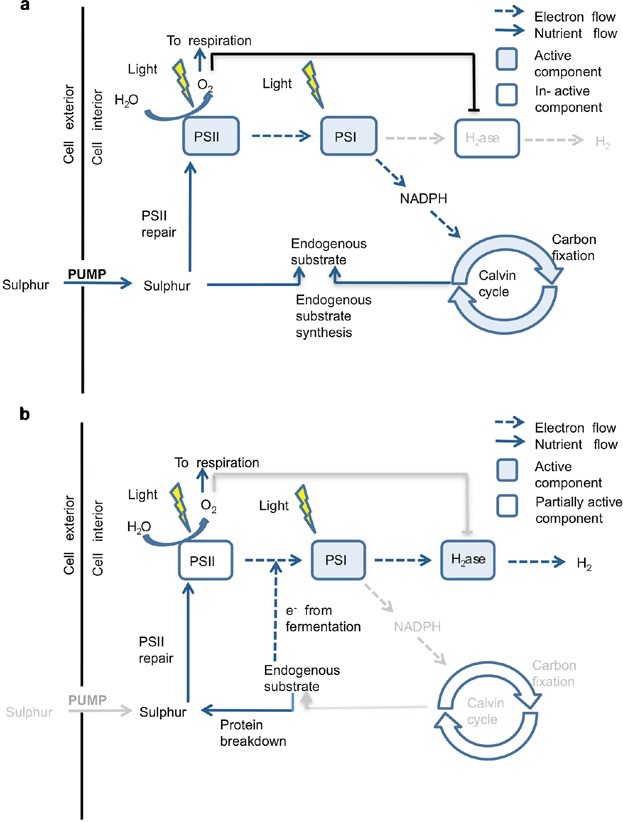
Schematics of the intracellular processes and pathways that occur under normal, sulfur-replete conditions (**panel a**) and during sulfur-deprivation (**panel b**). Light gray arrows and text indicate an inactive pathway/process. In panel (a), sufficient sulfur levels allow maximal PSII repair. Electron flow (dashed arrows) from PSII to PSI leads to ATP synthetase and oxygen production that inhibits the activity of the iron-hydrogenase (thick black line), where the Calvin cycle is active. Under sulfur-deprivation (panel b), PSII activity decreases, fermentation begins (releasing minimal quantities of sulfur and electrons) and low Calvin cycle activity, caused by Rubisco depletion, activates the iron-hydrogenase under anaerobic conditions.

Although the ability of the unicellular microorganism *Chlamydomonas reinhardtii* to photosynthetically produce hydrogen gas from water under illumination has been known for over 60 years (Gaffron and Rubin, 1942), until recently it remained largely a biological curiosity as hydrogen producing iron-hydrogenase is inhibited by oxygen co-produced from the photosynthetic pathway under normal illumination and nutrient conditions (Benemann et al., 1973; Ghirardi et al., 1997,2000). Thus photosynthetic growth and hydrogen production are incompatible and need to be spatially or temporally separated in order to achieve significant hydrogen production. Melis et al. (2000) proposed a groundbreaking two-stage process for temporally separating the hydrogen and oxygen components of the photosynthetic pathway: cells are grown as normal in a sulfur-replete media and then in a second non-growth stage, partial deactivation of the oxygen-evolving photosystem II (PSII) occurs in response to sulfur-deprivation. In essence, during water splitting in PSII, the sulfur-rich reaction-center D1 proteins are damaged and need to be replaced (Mattoo and Edelman, 1987). In the absence of sulfur, D1 protein biosynthesis is impeded and the PSII repair cycle is blocked (Wykoff et al., 1998), leading to a reduction in oxygen production to a low level (Melis et al., 2000). Aerobic respiration and the light-dependent activity of photosystem I (PSI) are not directly affected by sulfur-deprivation (Cao et al., 2001; Davies et al., 1994; Melis et al., 2000; Zhang and Melis, 2002). After approximately 24 h under illumination, the rate of oxygen produced from photosynthesis is less than the rate of oxygen consumed by respiration; in a sealed container, the cells consume dissolved oxygen in the medium and the culture becomes anaerobic (Ghirardi et al., 2000; Kosourov et al., 2002; Melis et al., 2000; Zhang et al., 2002). In addition, during this time electrons result from the catabolism of endogenous substrates such as protein and starch (e.g., Chochois et al., 2009; Fouchard et al., 2005; Posewitz et al., 2004), both of which have been shown to increase significantly in the initial stages of sulfur-deprivation before hydrogen is produced (Fouchard et al., 2005; Kosourov et al., 2002; Melis et al., 2000; Posewitz et al., 2004). These events cause morphological changes in the cells during hydrogen production (Zhang et al., 2002). During dark fermentation ethanol acts as an electron sink for any reducing equivalents produced, but ethanol is harmful to the cell (Kennedy et al., 1992). In the light, under sulfur-deprivation, the partially active respiratory chain does not suffice as an electron sink and nor does the Calvin cycle since Rubisco, a necessary sulfur-rich enzyme in carbon fixation, is broken down and not synthesized (White and Melis, 2006; Zhang et al., 2002). The oxygen sensitive iron-hydrogenase enzyme on the thylakoid membrane is activated under these conditions and steps in as a major electron sink, re-oxidizing potentially harmful electrons produced from both the PSII-dependent (via water splitting) and the PSII-independent (fermentation) pathways, yielding H_2_ gas for around 100 h in the light (Fouchard et al., 2005; Happe et al., 2002; Hemschemeier et al., 2008; Kosourov et al., 2002; Melis et al., 2000). The catabolic PSII-independent pathway is thought to contribute 20% of the hydrogen production and the PSII-dependent pathway contributes 80% (Fouchard et al., 2005; Volgusheva et al., 2013). Substantial hydrogen production ceases after around 120–140 h of sulfur-deprivation, thought to be due to depletion of the endogenous substrate available for catabolism (see Melis, 2002). Hence, there is a metabolic transition between an aerobic state with photosynthetic growth and an anaerobic state characterized by fermentation, H_2_ production and biomass reduction (Hemschemeier et al., 2008, see also Fig. 1). If sulfur is added to the culture once hydrogen production has ceased, the cells, and particularly PSII, can repair; cycles of oxygen production under *S*-sufficiency and H_2_ production under *S*-deprivation can result (e.g., Ghirardi et al., 2000).

The above description of the interplay between cellular processes is a simplification of very complex dynamics that whilst gaining general acceptance in the research community is subject to improvement (for recent reviews see Antal et al., 2010; Ghysels and Franck, 2010). Although the promising sulfur-deprivation protocol allows for significant hydrogen production, the efficiency of the two-stage process and the yields of hydrogen need to be improved to allow for commercial exploitation (see for example Das and Veziroglu, 2008; Melis, 2002). Scoma et al. (2012) demonstrated hydrogen production from green algae from solar light for the first time, but found that light conditions and mixing had a large effect on the H_2_ yield (see also Giannelli et al., 2009) (which is expected since the collective swimming behavior of such species is sensitive to light conditions, which in turn affects photosynthetic efficiency; Bees and Croze, 2010; Williams and Bees, 2011b). Furthermore, a large downtime arises due to sulfur-cycling between anaerobic sulfur-deprived hydrogen production and aerobic, sulfur-replete recovery periods. In order to advance beyond the standard two-stage process it is first necessary to understand the system within the limits of this procedure.

Strategies for the optimization of hydrogen gas production via the two-stage process can be designed and tested using dynamical models to represent the main pathways and processes of the system. To this end, we construct a simple mechanical, mathematical model of an algal culture that can describe sulfur-deprived hydrogen production in *C. reinhardtii* from a careful consideration of the biology and biochemistry, including important feedback pathways. The model is general in the sense that it captures both sulfur-deprived and sulfur-replete conditions. Beyond non-mechanistic approaches (Jo et al., 2006; Jorquera et al., 2008), there are two mechanistic models of aspects of the algal system under these conditions. Park and Moon (2007) constructed three separate state models of the biochemical photosynthetic processes involved in hydrogen production and specifically modeled eight primary metabolites. The release of hydrogen gas and the effects of illumination were explicitly modeled, but the role of endogenous substrates was omitted. Furthermore, the model is a discrete, multi-state model rather than a continuous formulation, and parameters values were difficult to identify. Fouchard et al. (2009) improved upon this approach by formulating a continuous description of the role of sulfur and light limitation in photosynthetic growth and anaerobiosis under general conditions and applied their model to the case of sulfur-deprivation, but the model stopped short of modeling the production of hydrogen gas. Model validation and optimization were considered by Degrenne et al. (2011).

In this study we shall improve upon previous work by modeling the principal mechanisms for the whole hydrogen production system, including feedback between sulfur uptake, photosynthetic growth, endogenous substrate, and the release of H_2_ gas. There are elements that are modeled in a similar fashion to Fouchard et al. (2009). In particular, both intra-and extra-cellular sulfur are considered and we describe the uptake of external sulfur using a modified Monod formulation (Monod, 1949) and illumination and photosynthetic activity are dynamically coupled, since it is well known that culture growth has an effect on the light available for photosynthesis. We describe the effects of sulfur-deprivation on the rate of photosynthesis using a similar modified-Droop relationship (Droop, 1968,1979), and the use of sulfur in PSII repair, and the release of oxygen from PSII and its consumption in respiration are also included. But, significantly, the current approach extends and refines previous work in a number of ways. Firstly, we model explicitly and mechanistically the initial storage and subsequent catabolism of endogenous substrate: protein breakdown in particular is important due to the release of small amounts of sulfur that can permit residual PSII activity, a key source of electrons for H_2_ production (e.g., Fouchard et al., 2005; Melis et al., 2000). We model substrate storage as dependent on the illuminated and *S*-dependent photosynthetic pathway (since proteins and starch are made via the Calvin cycle) and substrate breakdown (fermentation) as an emergency response to anaerobiosis, which also provides electrons to the hydrogenase. Culture growth can then be modeled as a function of endogenous substrate. These aspects differ from Degrenne et al. (2011) and Fouchard et al. (2009), since in these articles, changes in biomass are partitioned into growth and starch accumulation and these processes are not modeled independently (both depend in the same way on photosynthetic rate), protein dynamics and fermentation are not modeled explicitly, and feedback between substrate catabolism and sulfur release for PSII is not incorporated. The model could not adequately capture observed starch accumulation dynamics (Degrenne et al., 2011). The new description also deviates from the previous culture growth models in that it allows for both culture growth and biomass reduction under nutrient limitation (as shown experimentally in Zhang et al., 2002). Furthermore, it provides feedback pathways between growth, substrate catabolism and *S*-dependent photosynthesis. And finally, hydrogen production is modeled explicitly as a system output that is dependent on light and electron donation via both the PSII-independent and PSII-dependent pathways and is inhibited by oxygen within the culture (Ghirardi et al., 1997,2000).

The model presented here consists of a set of coupled ordinary differential equations driven by evolving culture conditions (see Williams, 2009). In the following sections, the model is constructed from a mechanistic perspective and the solutions explored numerically. As in previous publications in this area, parameter estimation and the determination of functional forms were considerable challenges. Our objective was to produce a robust mechanistic model that exhibits the same qualitative trends as observed in experiments, rather than to refine parameter values arbitrarily to obtain quantitative agreement. Parameter values or estimated ranges were obtained from published experimental studies (see “Supplementary Material”) and the model was then employed to probe the system subject to the constraints of the two-stage process outlined above. Model results are compared with published experimental data, and optimal external sulfur and illumination conditions are determined. In a subsequent paper, novel sulfur-cycling strategies will be explored for optimizing hydrogen production outside the confines of the two-stage process.

## Model Assumptions and Descriptions

### Model Formulation

The mechanistic model that we shall develop consists of a set of mass balance equations that represent three stages of hydrogen production: normal photosynthetic growth (Fig. 1a), activity under sulfur-deprivation and subsequent hydrogen production (Fig. 1b). We model an asynchronous cell population in a sealed, cubical container, purged of oxygen at *t* = 0 and filled with 1 L of culture, with illumination of 300 μE m^−2^ at two sides. Cell division and changes in individual cell size are combined into one variable, the cell volume fraction 0 ≤ *Λ* ≤ 1. We assume that oxygen diffusion across the cell wall is rapid, and thus internal and external oxygen concentrations can be described by one variable *ω*, in μM. External and internal sulfur, *S* and *s*, respectively, are not combined as they have distinct dynamics with active sulfur transport across the cell wall (Yildiz et al., 1994). Endogenous substrate, *e*, and protein *p* are also modeled explicitly. The variables *s*, *e*, and *p* are intracellular concentrations (or quota, Droop, 1968,1979), in μM: concentrations within the suspension in the bioreactor are given by, for example, *pΛ*. *S* is modeled as concentration in the suspension medium (μM; the concentration of external sulfur in the suspension is thus *S*(1 − *Λ*)). Hydrogen gas concentration, *h*, is modeled as a product in units of mL/L of culture, where we assume the same experimental and altitude conditions as Kosourov et al. (2002) such that 1 mL H_2_ = 33 μmol H_2_.

### The Effects of Culture Density on Light Availability

Cell volume fraction affects the optical density of the culture and thus the rate of photosynthesis. We model light intensity using the Beer–Lambert law (see Duysens, 1956), assuming that the cells are homogeneous and transmit light equally in all directions. The small effects of multiple scattering are neglected. Illumination is from the side, at *x* = 0, where 0 < *x* ≤ *b*_w_ measures the distance from the container edge to the light source of intensity *I*_0_ (*b*_w_ is bioreactor width). Assuming uniform cell concentration, *n*(*x*) = *n*_0_, light intensity is given by 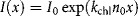, where for simplicity, the absorbance of the medium is assumed negligible and *k*_chl_ is the absorbance of the cells. Furthermore, we assume that the culture is well-mixed so that averaging over the width is the same as averaging over time:

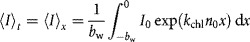
(1)

where 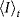 and 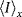 indicate time and space averages of *I*, respectively. These assumptions are incorrect if mixing is weak or swimming induced bioconvection results (Bees and Croze, 2010; Williams and Bees, 2011b). Furthermore, there is a light intensity, *I*_sat_ at which the photosynthetic rate saturates (Leverenz et al., 1990). Hence, imposing a Heaviside function and integrating (Supplementary Material; Williams, 2009), the dimensionless usable light, *L*(*Λ*), is

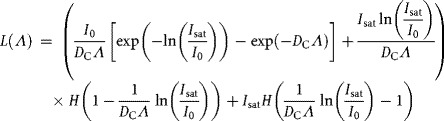
(2)

where we define 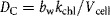. Here, *L*(*Λ*) has been normalized with *L*_*e*1_ and the light intensities are non-dimensionalized with *L*_*e*1_ (e.g., 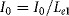), a standard value employed in Kosourov et al. (2002) subject to which other parameters are measured and inferred (tildes have been dropped, see Table I and Supplementary Material).

**Table I:** Table of standard model parameters. The range of values is calculated using errors/ranges from data or by simply estimating (denoted as ^*^)

Notation	Parameter	Value	Unit	Range	Reference
*a*	Rate constant for *S* uptake over normal cell volume	14,800	μM h^−1^	12,500–17,100	Yildiz et al. (1994)
*b*_1_	Rate constant for sulfur uptake	2.2	μM	1.3–3.1	Yildiz et al. (1994)
*b*_2_	Rate constant for sulfur uptake	14.5	N/A	14.5–19.8	Yildiz et al. (1994)
*b*_ω_	Width of the bio-reactor	10.0	cm	1–100	N/A
*E*_L_	Fraction of electrons from PSII-dependent path	0.75	N/A	0.7–0.8	Fouchard et al. (2005)
*G*	Dimensionless scale factor	2.29	N/A	1.77–2.99	Yildiz et al. (1994)
*I*_sat_	Non-dimensional light saturation	24.8	N/A	20–30.0	Leverenz et al. (1990)
*I*_0_	Non-dimensional light intensity at the source	99.2	N/A	0.0–200.0	Kosourov et al. (2002)
*k*_1_	Rate constant for PSII repair	0.041^*^	h^−1^	0.376–0.451^*^	Kosourov et al. (2002), Melis et al. (2000)
*k*_2_	Rate constant for protein breakdown	0.08^*^	h^−1^	0.0267–0.0973^*^	Kosourov et al. (2002)
*k*_3_	Rate constant for protein production	56.4^*^	μM h^−1^	51.7–61.1^*^	Kosourov et al. (2002), Melis et al. (2000)
*k*_4_	Rate constant for hydrogen production	773.0	mL h^−1^	595.0–1068.0	Kosourov et al. (2002)
*k*_5_	Rate constant for oxygen consumption by respiration	26,40,00.0	h^−1^	247,000–281,000^*^	Kosourov et al. (2002)
*k*_6_	Rate constant for oxygen production from PSII	12,400,00.0	μM h^−1^	1,000,000–1,480,000^*^	Kosourov et al. (2002)
*k*_chl_	Measure of absorbance of the cells	1.32 × 10^−6^	cm^2^	(1–3) × 10^−6^^*^	Berberoglu et al. (2008)
*L*_*e*1_	Normalization value for useable light	6.05	μmol m^−2^ s^−1^	N/A	Kosourov et al. (2002)
*p*_0_	Protein level when growth is zero	1370.0	μM	1240–1770	Kosourov et al. (2002)
*p*_1_	Protein below which maximum decay occurs	1350.0	μM	1180–1690	Kosourov et al. (2002)
*p*_2_	Protein required for maximum growth	1,570.0	μM	1480–1650	Kosourov et al. (2002)
*p*_r_	Basic protein needed for cell survival	206.0	μM	100–300^*^	Kosourov et al. (2002)
*p*_h_	Normalization of PSII-independent electron pathway	1,260	μM	1000–1400^*^	Kosourov et al. (2002)
*r*_exp_	Maximum growth rate	0.064	h^−1^	0.037–0.064	Fischer et al. (2006), Jo et al. (2006), Kosourov et al. (2002)
*r*_decay_	Maximum rate for cell decay	0.0053	h^−1^	0.001–0.01^*^	Kosourov et al. (2002)
*s*_n_	Normal level of sulfur in a cell	15,000^*^	μM	10^3^–10^5^	Hiriart-Baer et al. (2006)
*s*_1_	Sulfur level above which Calvin cycle is active	7,500^*^	μM	3000–15,000^*^	Zhang et al. (2002)
*s*_h_	Normalization of PSII-dependent electron pathway	2,500	μM	1,250–3,750^*^	Kosourov et al. (2002)
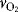	Oxygen mass transfer coefficient	0.374	N/A	0.03–0.5	Mölder et al. (2005)
*β*	Average moles of sulfur in 1 mol of protein	0.5^*^	N/A	0.1–15.0	Goldschmidt-Clermont and Rahire (1986), Thompson et al. (1995)
χ	Oxygen saturation in water	253.0	μM	200–300	Lewis (2006); Weiss (1970)
*ω*_1_	Oxygen required for full respiration	1.18	μM	0.75–2.0	Forti and Caldiroli (2005)
*ω*_2_	Oxygen level with prevents *H*_2_ production	26.0	μM	13–39^*^	Flynn et al. (2002)
*ω*_p_	Oxygen level below which protein breakdown occurs	26.0	μM	13–39^*^	Flynn et al. (2002)

“Reference” refers to the publication from which the parameter was collected or derived (see Supplementary Materials for full details).

### Sulfur Kinetics

We employ data from Yildiz et al. (1994), to model the uptake of external sulfur into the cells from the media: sulfur uptake is dependent on both external and internal sulfur concentrations (uptake rate varied between sulfur-starved and normal cells; also shown experimentally in Fouchard et al., 2009). This leads to a modified Monod formulation for the total sulfur uptake for cell volume fraction *Λ* in which the Michaelis-Menten uptake rate under normal, s-replete conditions, *α*(*s*), and the half saturation value, *β*(*s*), are in this case sulfur dependent functions:

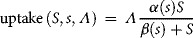
(3)

Assuming that *s* = 0 in the starved cells and that *s* = *s*_n_, the “normal” amount of sulfur, within an unstarved cell then we fit 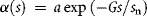, 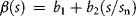 to the data in Yildiz et al. (1994), where *a* is the maximum uptake rate of external sulfur (values shown in Table I). Thus for total external sulfur in the media, *S*(1 − *Λ*), we obtain

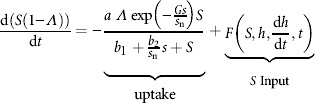
(4)

where *F* is an arbitrary addition of external sulfur to the bioreactor, which may depend on external sulfur, *S*, hydrogen, *h*, rate of hydrogen production, d*h*/d*t*, and time.

Inside the cell, sulfur is used in replacing photo-damaged PSII (termed “repair” herein) and in making other proteins. We assume the use of sulfur for PSII repair is linearly dependent on light, due to photo-damage, and available sulfur. A Heaviside switch function *H*_PSII_ denotes that above a critical concentration of internal sulfur, *s*_n_, photosynthetic activity is not affected by *s* concentration, and photosynthesis and thus PSII repair occurs at a constant rate:

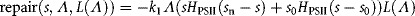
(5)

This relationship is analogous to the modified Droop formulation with a switch function employed by Degrenne et al. (2011) and Fouchard et al. (2009) in which PSII activity drops off rapidly once sulfur falls below the critical quota value. Encouragingly, the corresponding curve of photosynthetic activity as a function of *s* agrees with the experimental measurements of Fouchard et al. (2009) (not shown).

Intracellular protein concentration *p* is a large component of endogenous substrate and can act as a sulfur store: during anaerobic fermentation protein is catabolized to release sulfur (Melis et al., 2000). We model this sulfur source as dependent on available protein and oxygen levels using a switch function to specify that fermentation only occurs during anaerobiosis, *ω* < *ω*_p_ (Happe et al., 2002). A non-consumable base level of protein, *p*_*r*_, necessary for cell survival is also modeled (shown experimentally in Kosourov et al., 2002)


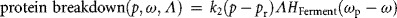
(6)

**Figure 2:**
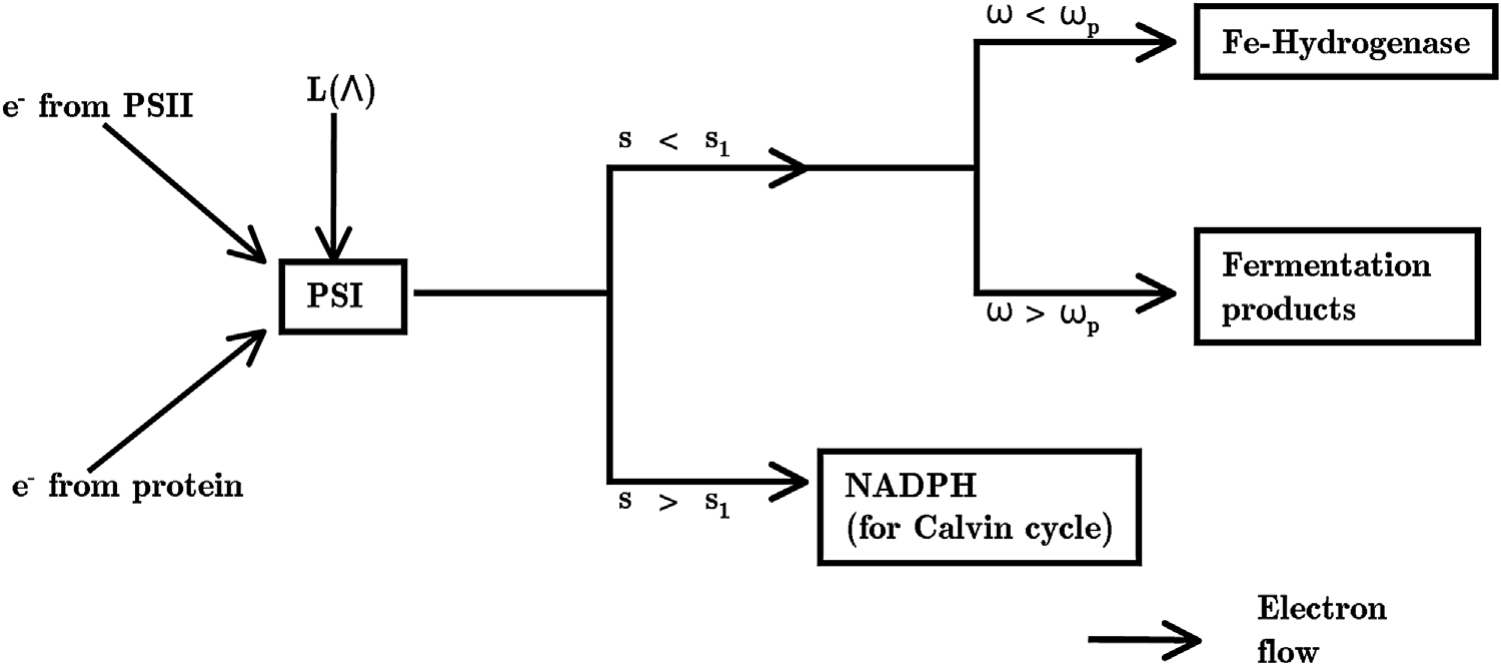
A diagram of the transport of electrons from the PSII-dependent pathway and the PSII-independent pathway to PSI and on to either the iron-hydrogenase, fermentation products or the Calvin cycle. Where the electrons end up is decided by the oxygen and sulfur dependence, as indicated.

Protein is produced under normal conditions, combining sulfur with carbon skeletons produced from the photosynthetically dependent Calvin cycle. Thus we model protein production linearly on sulfur availability and light intensity up to a certain concentration of *s*, *s*_n_, using *H*_PSII_ normalized with *s*_n_ to stipulate that given sufficient sulfur, photosynthetic activity is constant (see Equation 5). We assume that the cell can use one of the Calvin cycle, ethanol production or H_2_ production as an electron sink at any one time (see Fig. 2). This assumption is realized by using a switch function 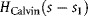 to stipulate that protein is only produced when sufficient sulfur (*s* > *s*_1_), thus sufficient Rubisco, allows the Calvin cycle to function (White and Melis, 2006; Zhang et al., 2002) (see Disussions and Conclusions section). Thus the model of internal *s* and *p* concentrations is

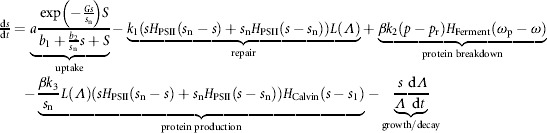
(7)


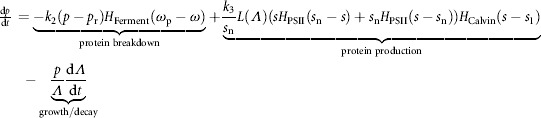
(8)

where *β* indicates that 1 mol of protein contains *β* moles of sulfur. The PSII repair term does not appear elsewhere in the model, implying that sulfur used in PSII repair is not recycled. Note the growth terms in Equations (7) and (8), which arise as increasing cell volume alone reduces concentration.

### Oxygen Kinetics

Under normal conditions PSII produces oxygen and respiration consumes oxygen. The relationship between PSII activity and sulfur is given in Equation (5) (for the rate of sulfur consumption) and is used here but with oxygenic photosynthetic rate constant *k*_6_ (μM O_2_ h^−1^) and the non-dimensional PSII-switch. Respiration rate remains relatively unaffected by sulfur-deprivation (for *t* < 70 h; Melis et al., 2000) and thus is modeled as constant when oxygen is sufficient, *ω* > *ω*_1_, but decreases linearly when oxygen is sparse, *ω* < *ω*_1_. Although the bio-reactor is sealed, we stipulate that O_2_ can leave the system when the culture is oxygen saturated and cannot reenter. Thus

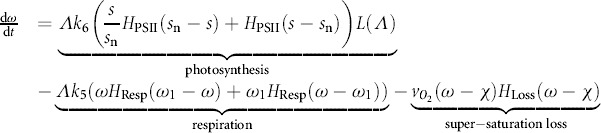
(9)

### Growth and Decay of Cell Culture

Changes in cell volume fraction can occur due to both photosynthetic biomass production and substrate breakdown. Since endogenous substrate is explicitly modeled, we can incorporate changes in culture concentration dependent on endogenous substrate rather than on the rate of photosynthesis (as modeled in Fouchard et al., 2009) to allow for both growth and decay of the culture (Zhang et al., 2002). Under sulfur sufficiency the culture density increases as endogenous substrate increases, whereas under nutrient deprivation substrate breaks down and the culture density decreases. Changes in endogenous substrate are largely due to protein and starch, both of which initially increase and then decrease during H_2_ production (e.g., Degrenne et al., 2011; Fouchard et al., 2005; Kosourov et al., 2002). We assume that protein and starch are sufficiently correlated to allow modeling them as one entity, and thus we model growth rate as explicitly dependent on protein (*e* = *p* from here on).

The growth function is chosen so that the growth and decay rates are constant above and below, respectively, critical levels of protein (*p*_2_ and *p*_1_, respectively, see Table I for details); and there is some linear transition between the two states, giving an “s” shaped function (using a smoothed version had no qualitative effect). Light dependence is modeled explicitly in protein production and thus is implicit in the growth term. Thus

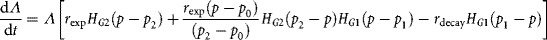
(10)

where *r*_exp_ and *r*_decay_ are the maximum growth and decay rates, respectively.

### Hydrogen Production

Hydrogen production is modeled as dependent on the scaled sum of electrons coming from endogenous substrate catabolism (the PSII-independent pathway, dependent on *p*) and from the residual level of the PSII activity (the PSII-dependent pathway, dependent on *s*) (Fouchard et al., 2005; Happe et al., 2002). These pathways are assumed independent and, as for growth, we assume protein and starch catabolism act in the same way and base our model on protein concentration alone. The Calvin cycle switch function *H*_Calvin_(*s*_1_ − *s*) is used to stipulate that the Calvin cycle also needs to be inactive for the hydrogenase to function as the electron sink (see Fig. 2), and an oxygen sensitive switch is employed to reflect the dependence of iron hydrogenase on anaerobic conditions. Thus

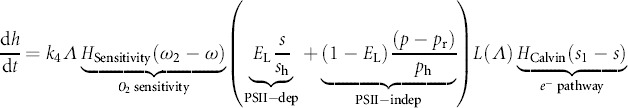
(11)

where *E*_L_ is the fraction of electrons from the PSII-dependent pathway under total sulfur-deprivation (see Table I and Supplementary material for details).

Thus mass balance equations (4) and (7)–(11) make up the standard model. Parameters definitions, values, and references are summarized in Table I. Parameters are taken from the literature where possible or else estimated using available relevant data (see Supplementary Material for full details). The model is non-dimensionalized using 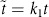, 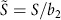, 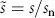, 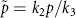, 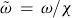, and 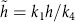. The scaling for time is chosen so that one non-dimensional time unit corresponds to approximately 1 day. The sulfur scaling is chosen so that *s* = 1 initially (under normal conditions). The non-dimensional standard model equations and parameters are shown in Appendix A (tildes are dropped from here on).

## Results

To illustrate the model dynamics the set of differential equations were solved numerically using Matlab 7.0 software (R2007) with the robust implicit scheme “ode15s” (employing a modified backward Euler method; Shampine and Reichelt, 1997). The numerical method was verified with a known solution for a simplified and linearized version of the model. The initial conditions were chosen to be representative of experimental conditions in Kosourov et al. (2002): *S* = 1, *ω* = 0, *p* = 2.23, *Λ* = 2.25 × 10^−3^, *h* = 0, and initial external sulfur concentration *S*_0_ varied between model runs. For numerical simulations, approximations to continuous functions are preferred over discontinuous (Heaviside) switches (as in Degrenne et al., 2011). Thus hyperbolic tangent switches were thus employed:

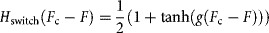
(12)

This function of *F* varies rapidly from 1 to 0 around the critical value *F*_c_ for a large value of the parameter *g*; it was increased to a value beyond which it did not significantly affect model output (Williams, 2009).

**Figure 3:**
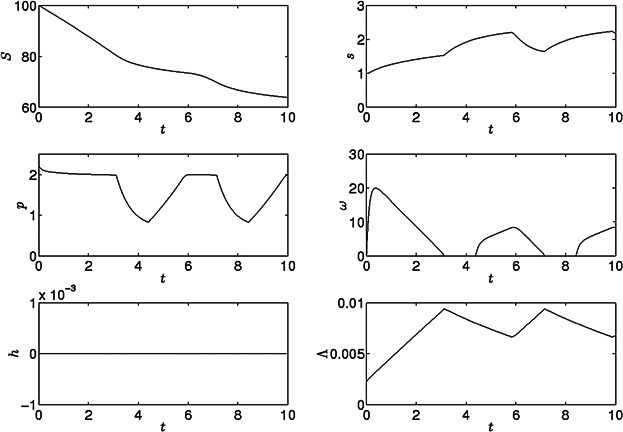
Results for the model with standard parameter values in Table I under sulfur-replete conditions, *S*_0_ = 100 (non-dimensional units).

Results with a large cell concentration in a sulfur-replete medium in sealed conditions are shown in Figure 3. There is a rapid increase in cell volume fraction in the first 3 days, with a doubling time of 22–23 h, compared to 9.4–18.6 h calculated from experimental data (Fischer et al., 2006; Jo et al., 2006). After 3 days, light limitation decreases oxygen production from PSII, resulting in anaerobic fermentation. Hydrogen production is not observed as sufficient sulfur is available for the Calvin cycle (via Rubisco) to act as the electron sink. Fermentation causes cell volume fraction to decrease, decreasing light limitation and, since sulfur is available, PSII activity increases and the system subsequently becomes aerobic and a period of protein production and growth follow. Thereafter, oscillations in *s*, *p*, *ω*, and *Λ* are found (period around 97 h), with no hydrogen produced. These results are consistent with Zhang et al. (2002), in which a concentrated culture in a sealed container became anaerobic as cell density increased, but only inactive hydrogenase was found. A bioreactor culture could be continuously diluted to optimize growth and avoid over-densification (see also Fouchard et al., 2009).

**Figure 4:**
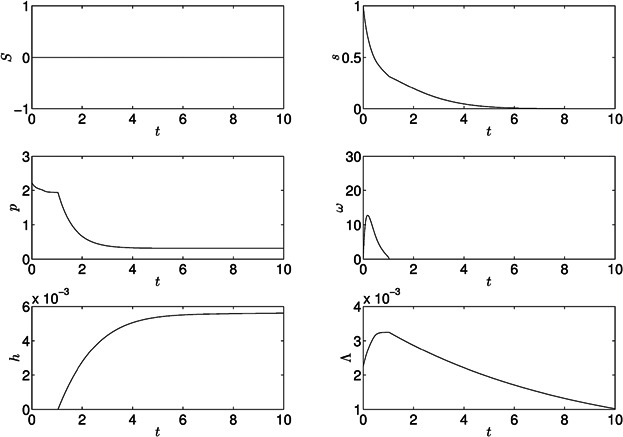
Results for the model with standard parameter values in Table I under sulfur-deprivation, *S*_0_ = 0.

Figure 4 shows the model results for a culture suspended in a sulfur-free media, *S*_0_ = 0 μM, at *t* = 0. The model is run for approximately 10 days. Internal sulfur immediately starts to decrease while cell volume fraction increases initially as sulfur is still sufficient for Calvin cycle activity and growth, *s* > *S*_1_. When *s* falls below *S*_1_ growth slows down as the Calvin cycle becomes inactive due to a lack of sulfur and thus protein is not produced. As *s* decreases further, the oxygenic photosynthetic rate falls below the respiration rate and a period of anaerobiosis begins after approximately 1 day. Since the Calvin cycle is also inactive under *s*-deprivation, hydrogen production now commences, and fermentative protein breakdown begins, resulting in release of small amounts of internal sulfur. *p* and *Λ* decrease during this H_2_ production phase due to catabolism of endogenous substrate. *p* reaches *P*_R_, the base level of protein needed for cell survival, between 2 and 4 days, but protein breakdown continues to supply electrons and sulfur to the photosynthetic pathway because the shrinking cell volume fraction causes oscillations in *p* around *P*_R_ (as *Λ* decreases, cellular protein concentration *p* increases transiently; total protein in the culture, *pΛ*, monotonically decreases). The initial hydrogen production rate is rapid but decreases significantly at around 6 days, when internal sulfur has run out, PSII activity stops, endogenous substrate is low and only minimal amounts of hydrogen are now produced from the PSII-independent pathway. After 2 more days hydrogen production stops and the cells continue to shrink. The final yield of gas after ten days is 106 mL H_2_/L culture, and after 140 h (*t* = 5.74) is 103 mL H_2_/L culture compared to 71.7 mL H_2_/L culture in 140 h in Kosourov et al. (2002). Results for *s*, *ω* and *Λ* are qualitatively similar to model results by Fouchard et al. (2009).

### Optimizing H_2_ Yield: Varying Initial External Sulfur, *S*_0_

**Figure 5:**
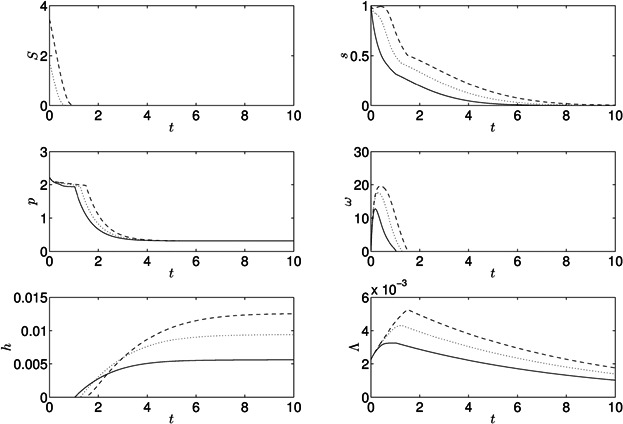
Results for the model with standard parameter values in Table I, with initial conditions of external sulfur of *S*_0_ = 0 (solid lines), *S*_0_ = 1.725 (dotted lines), and *S*_0_ = 3.45 (dashed lines). These correspond to 0, 25, and 50 μM, respectively.

**Figure 6:**
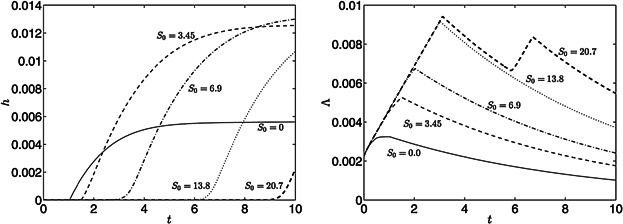
Hydrogen and cell volume fraction curves for the model with standard parameter values in Table I and with initial conditions *S*_0_ = 0 (solid lines), 3.45 (dashed lines), 6.9 (dot-dashed lines), 13.8 (dotted lines), and 20.7 (thick dashed lines) in non-dimensional units.

Re-suspending the cells in media with minimal rather than zero concentrations of external sulfur has been shown to increase the total yield of hydrogen gas (Kosourov et al., 2002; Zhang et al., 2002). Figure 5 shows model results for different initial concentrations of external sulfur, *S*_0_. For *S*_0_ > 0 internal sulfur and protein decrease slower than when *S*_0_ > 0, leading to higher culture density. Increased oxygen combined with a later decay in *p* and *s* leads to a later onset of anaerobiosis and a delay between this onset and hydrogen production. For *S*_0_ = 3.45 (50 μM), yields of hydrogen gas are significantly larger than for *S*_0_ > 0 at *t* = 10 (*h* = 237 mL H_2_/L vs. *h* = 106 mL H_2_/L, respectively), and production begins later (*t* = 45.2 h when *S*_0_ = 3.45 and *t* = 36.4 h when *S*_0_ = 1.725). Figure 6 shows these results in detail: increasing *S*_0_ from zero to *S*_0_ = 6.9 delays the onset of H_2_ production and increases yield at *t* = 10 but, as *S*_0_ is increased further, yields decrease until hydrogen is not produced in this time frame. The optimal *S*_0_ for H_2_ yield at *t* = 10 is *S*_0_ = 6.19 (89.8 μM) with *h* = 246 mL H_2_/L culture.

**Figure 7:**
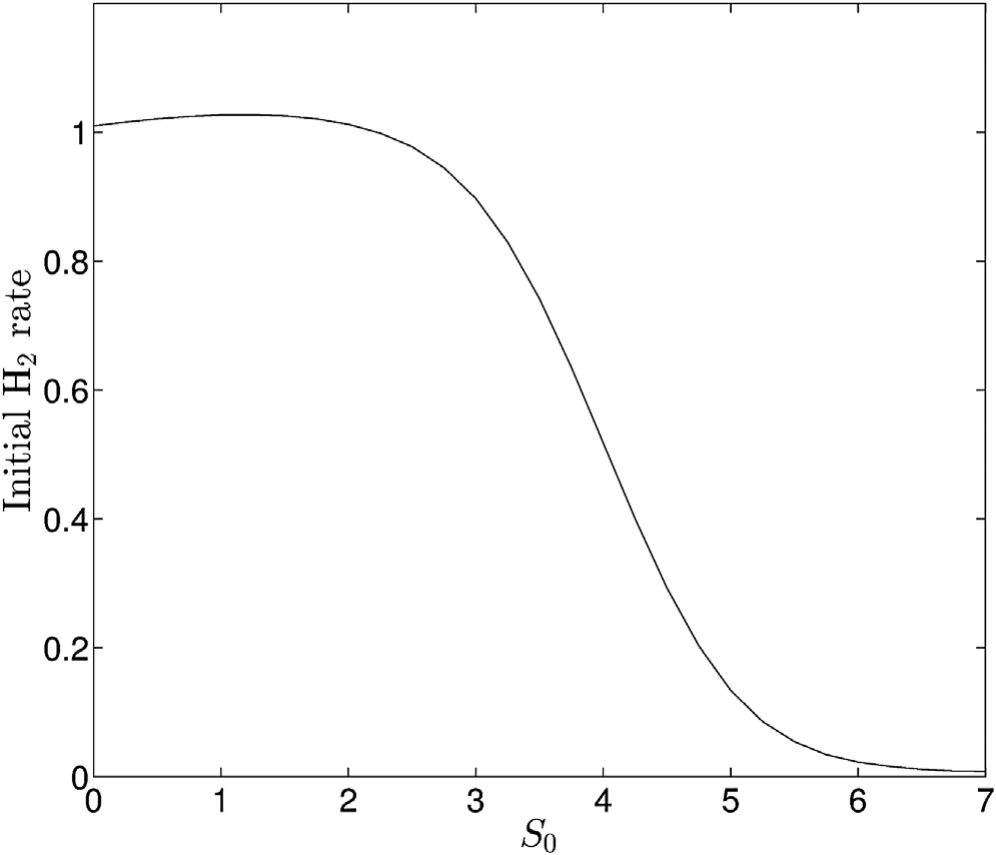
Initial rates of hydrogen production (in the first 15 h) plotted against the initial amount of external sulfur for the standard parameter values in Table I.

The average initial rate of H_2_ production over the first 15 h of production is calculated per unit of cell volume fraction using

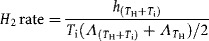
(13)

where *T*_H_ is the onset time of hydrogen production, *T*_i_ = 0.6 is the scaled initial time-period of production considered, and *Λ* is averaged over the initial hydrogen production period. Figure 7 shows a slight increase in the initial rate of hydrogen production per cell volume fraction as *S*_0_ is increased from zero up to *S*_0_ ≈ 1.25, and thereafter the H_2_ production rate decreases and reaches very low levels at *S*_0_ = 6. Thus there is an optimal initial value for external *S*_0_ for improving the rate of hydrogen produced per cell (*S*_0_ ≈ 1), which is different to the optimal for improving yield at time *t* = 10 (*S*_0_ = 6.19).

### Optimizing H_2_ Yield: Varying Light Intensity, *I*_0_

**Figure 8:**
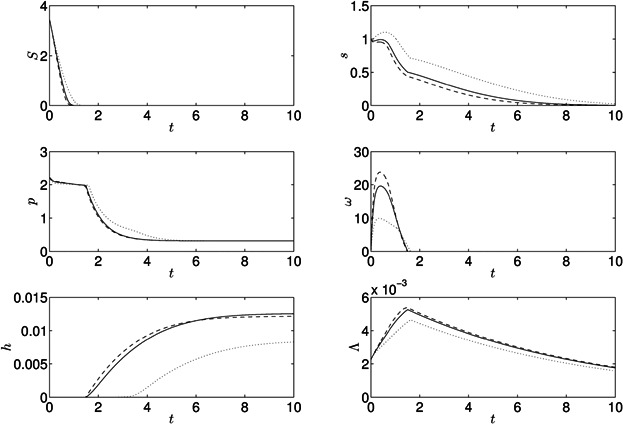
Model results when *I*_0_ is increased (dashed lines) and decreased (dotted lines) by a factor of 2, compared to model results for *S*_0_ = 3.45 and the standard parameter values in Table I (solid lines).

**Figure 9:**
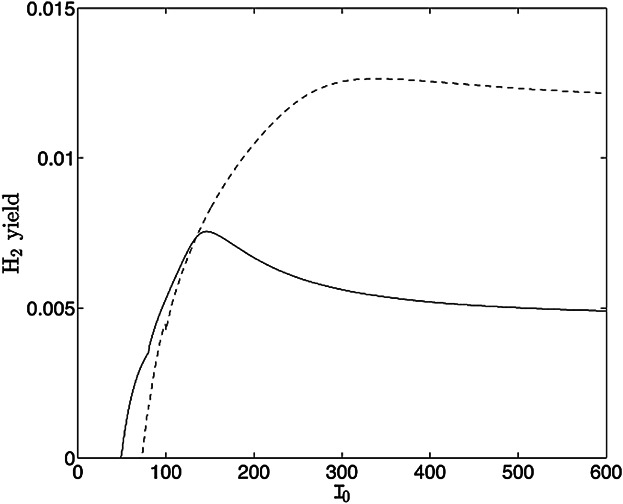
Hydrogen yield at *t* = 10 as a function of dimensional *I*_0_ when *S*_0_ = 0 (solid line) and *S*_0_ = 3.45 (dashed line) for the standard parameter values in Table I.

Varying the illumination conditions of the algal culture can have an effect on the yield of hydrogen gas (e.g., Degrenne et al., 2011; Kim et al., 2006). Figure 8 shows results for three values of the light intensity, *I*_0_ when *S*_0_ = 3.45 (50 μM). For *I*_0_ = 49.6 (half of the standard value), slower growth and, hence, slower *s* usage delay onset of H_2_ production. A smaller cell volume fraction combined with reduced activity of PSII-dependent activity result in a decreased H_2_ yield, as expected. When *I*_0_ is doubled from the standard value, *I*_0_ = 198.4, rapid growth leads to higher cell volume fraction and faster sulfur usage compared to the standard case. However, the resulting increase in oxygen production causes the system to become anaerobic and hence produce H_2_ at approximately the same time. Perhaps surprisingly, hydrogen production stops sooner and the yield is significantly reduced with higher *I*_0_ even though the cell density and light available for the PSII-dependent electron pathway have increased. This is due to increased PSII photo-damage causing a more rapid decline in internal sulfur, which limits PSII-dependent electron donation. Thus, there is an optimal light intensity to maximize hydrogen yield within a given time (as shown experimentally by Kim et al. (2006)): for the model presented here, for *t*_end_ = 10 the optimal light intensity is *I*_0_ = 146.5 μmol m^−2^ s^−1^ if *S*_0_ = 0 and *I*_0_ = 340 μmol m^−2^ s^−1^ if *S*_0_ = 3.45 (50 μM: see Fig. 9). Thus the initial sulfur concentration has an effect on the optimal light intensity. Decreasing the cellular absorption coefficient, *D*_C_, provides more light on average to each cell and thus also results in a greater hydrogen yield: for *I*_0_ = 300 μmol m^−2^ s^−1^, decreasing *D*_C_ increases yield. However, for large light intensity *I*_0_ and small *D*_C_, the effects of photo-damage cause an overall decrease in yield.

## Discussion and Conclusions

A simple mechanistic model has been constructed to describe sulfur-deprived hydrogen production in green algae. By modeling mechanistically, we have significantly simplified this complex system to just six variables. Key features of the model, including sulfur-dependent photosynthesis, growth, changes in endogenous substrate, and hydrogen gas release, have been incorporated. Solutions were obtained for the standard values of the parameters and with a range of initial conditions.

The experimental studies of Kosourov et al. (2002), Melis et al. (2000), and Zhang et al. (2002) guided the construction of the model and some parameters in the growth and hydrogen functions, in particular, were extrapolated from the experiments therein. For example, the hydrogen production rate constant *k*_4_ was taken from measured data in Kosourov et al. (2002) for the case of *S*_0_ = 0 μM, thus the simulated hydrogen dynamics match the experimental data for *S*_0_ = 0 μM reasonably well, as expected. However, the model was not fit to the data, and the hydrogen dynamics for different initial external sulfur and illumination conditions can still be compared independently with experimental data in order to test whether the model correctly captures the system dynamics under different conditions. Better independent measurements of the parameters, rather than fitting, should be the focus of future research efforts. Encouragingly, good qualitative and quantitative agreement was obtained between experimental results and model simulations for H_2_ yield for different initial external sulfur concentrations, *S*_0_: after 140 h if *S*_0_ = 25 μM or *S*_0_ = 50 μM, the model predicts yields of *h* = 168 mL H_2_/L culture and *h* = 213 mL H_2_/L culture, respectively, in good agreement with Kosourov et al. (2002) (*h* = 127 and *h* = 159 mL H_2_/L culture, respectively). The optimal *S*_0_ for maximum hydrogen output over a fixed period was found to be a dynamic balance between high culture density, light limitation, and production start time. Hydrogen production onset time also corresponded approximately to experimental results: for *S*_0_ = 25 μM and *S*_0_ = 50 μM, *t* = 36.4, and *t* = 45.2 h, respectively, compared to *t* = 43–49 h in Kosourov et al. (2002). In simulation results, hydrogen production began almost as soon as the system became anaerobic when *S* = 0 μM, as in Zhang et al. (2002), but Kosourov et al. (2002) found a slight delay between onset of anaerobiosis and hydrogen production. This delay was predicted by our model for *S* > 0 μM, due to slower sulfur decay causing an extended period of Calvin cycle activity.

The initial rate of hydrogen production per cell was also investigated, and we found that it increased slightly then decreased substantially as *S*_0_ increased. The relatively constant production rate per cell is consistent with experimental observations from Degrenne et al. (2011) and Zhang et al. (2002) (where rate is per gram of biomass), but inconsistent with Kosourov et al. (2002), who found an increase in initial H_2_ production rate per mole of chlorophyll for *S*_0_ = 25 μM compared to *S*_0_ = 0. We attribute increased hydrogen yield for *S*_0_ ≈ 50 μM to increased cell volume fraction, as found experimentally by Zhang et al. (2002), rather than increased production rate per cell as proposed by Kosourov et al. (2002). The decrease in the initial rate for *S*_0_ > 43.5 μM found from our model is also consistent with the trends found by Kosourov et al. (2002) and Zhang et al. (2002) for *S*_0_ ≥ 25 μM. Likewise, we attribute corresponding decreases in H_2_ yield to increased light limitation counteracting further increases in *Λ* when *S*_0_ is large. The optimal sulfur concentration for maximizing this H_2_ production rate (approximately 0 ≤ *S*_0_ ≤ 29 μM) was found to be different from the optimal sulfur for increasing overall yield (*S*_0_ = 89.9 μM). Thus methods of optimization of the hydrogen production system depend on whether maximum cell activity or maximum H_2_ output per culture is required.

Model simulations for changes in illumination are consistent with experimental data from Hahn et al. (2004) and Kim et al. (2006): increasing the light intensity *I*_0_ can significantly increase yields up to an optimal value due to earlier onset of production and increased culture density and PSII-dependent electron flow. However, increasing *I*_0_ beyond the optimal value decreases H_2_ yields due to increased photo-damage, as in Kim et al. (2006). Simulation results predict an optimal light intensity for total H_2_ output of *I*_0_ = 146.5 μmol m^−2^ s^−1^ for *S*_0_ = 0 μM or *I*_0_ = 340 μmol m^−2^ s^−1^ for *S*_0_ = 50 μM with illumination from both sides, which are of the same order as those predicted by Park and Moon (2007) (238 μE m^−2^ s^−1^) and Kim et al. (2006) (200 μE m^−2^ s^−1^).

Using the model of Degrenne et al. (2011), Fouchard et al. (2009) found qualitatively similar results: they predicted that a high hydrogen yield would require high external sulfur and light irradiance. Experimental data supported this conclusion. However, in those studies H_2_ gas production was not explicitly modelled but was extrapolated from biomass and starch concentrations. We find that higher yields of H_2_ are found for higher cell volume fraction: for *S*_0_ = 0, *h* = 247 mL H_2_/L with *Λ*_0_ = 0.0045 and 106 mL H_2_/L culture when *Λ*_0_ = 0.00225, which supports the hypothesis that one may optimize H_2_ yield by maximizing biomass. However, we caution that for sufficiently high initial sulfur and light conditions our model predicts diminished H_2_ yields due to over-concentrated cultures and photo-damage.

Melis (2002), Melis (2009), and Polle et al. (2002) suggested that truncating the chlorophyll antenna to decrease cellular absorbance (modelled as *D*_C_) decreases wasted light and increases photosynthetic activity, which may increase the hydrogen yield. Model results also suggest that decreasing absorbance could optimize H_2_ yield, provided that the light intensity *I*_0_ is not too high, or *D*_C_ is not too low, otherwise yields decrease due to increased photo-damage (as for high light intensities in this model and in Kim et al. (2006) and Park and Moon (2007).

To our knowledge, this is the first simple mechanistic model of sulfur-deprived hydrogen production to include feedback between sulfur, photosynthetic growth, endogenous substrate, and hydrogen production. Good qualitative agreement is found between model simulations and experimental results. In order to model such a complex system, key assumptions were made. The role of starch was not modelled independently; instead, endogenous substrate is representative of both protein and starch in order to capture the dynamical feedback between sulfur, photosynthetic growth and fermentation. This may be a reasonable approximation, but the two may be better modelled separately, with growth a function of both. However, we do not expect this extension qualitatively to alter results.

Additionally, a switch (*H*_Calvin_ (*s*; *S*_1_)) was used to close the system and specify that H_2_-producing hydrogenase requires both anaerobiosis and an inactive Calvin cycle to function as an electron sink (e.g., Happe et al., 2002; Hemschemeier et al., 2008; White and Melis, 2006), so a sealed system with high culture density leads to anaerobiosis due to light limitations but no hydrogen is produced (in accordance with Zhang et al., 2002). In this study, the switch had little effect when initial external sulfur was minimal and it allowed the omission of the complex Calvin cycle and the interplay between electron sinks from the model. It may be revealing to explore further the explicit nature of the coupling between the hydrogenase and the Calvin cycle.

To describe the suspension, the cultures were assumed to be perfectly mixed and cell swimming behaviour was not described. Biased swimming is known to induce hydrodynamic instabilities, resulting in non-uniform distributions of cells, called bioconvection, in tens of seconds on length scales of centimeters. This significantly affects light transmittance and thus photosynthesis (Bees and Croze, 2010; Williams and Bees, 2011a,2011b), and could have a substantial impact on H_2_ yield. All of these assumptions should be explored in future developments of the current model.

As new data emerge, refinements of the parameter values and key mechanisms can be incorporated in the model. Perhaps more importantly, the current description is ideal for examining novel regimes for optimizing the total yield, or rates of production, of hydrogen gas produced under a range of sulfur-deprivation schemes. Such analysis may provide valuable insight into future commercialization of algal H_2_ production and will be presented in a future article.

## Figures and Tables

**Table II:** Expected range of non-dimensional parameter values, calculated from Table I

Dimensionless parameter	Definition	Description	Standard value	Range
*A*_1_	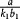	Scaled ratio of sulfur uptake rates	1.64 × 10^5^	8.94 × 10^4^–3.5 × 10^5^
*A*_2_	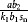	Scaled ratio of sulfur uptake rates	159.0	9.39–6480
*B*	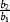	Ratio of *S* uptake rates	6.65	3.39–14.2
*D*_C_	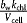	Scaled measure of absorption	26,300	2 × 10^3^–6 × 10^5^
*E*_L_	*E*_L_	Fraction of electrons from PSII-dependent path	0.75	0.7–0.8
*I*_sat_	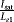	Normalized light saturation	24.8	20–30.0
*I*_0_	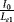	Normalized light intensity at source	99.2	0.0–200.0
*K*_2_	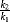	Scaled protein breakdown rate	1.95	0.592–2.59
*K*_3_	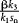	Scaled measure of rate of *p* production	0.0459	0.0057–25.4
*K*_5_	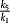	Scaled respiration rate	6.44 × 10^6^	5.48 × 10^6^–7.47 × 10^6^
*K*_6_	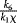	Scaled photosynthesis rate	1.2 × 10^5^	7.39 × 10^4^–1.97 × 10^5^
*P*_G_	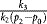	Scaled protein gradient	3.52	0.699–5.9
*P*_H_	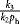	Non-dimensional reciprocal of *p*_h_	0.560	0.380–2.29
*P*_R_	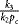	Scaled protein required for survival *p*_r_	0.292	0.0437–0.565
*R*_G_	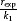	Scaled growth rate	1.56	0.82–2.39
*R*_D_	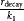	Scaled decay rate	0.129	0.0221–0.266
*S*_1_	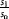	Ratio of *s* required for Calvin cycle to normal *s*	0.5	0.2–1.0
*S*_H_	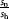	Non-dimensional reciprocal of *s*_h_	6.0	4.0–12.0
*V*_L_	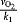	Scaled oxygen mass transfer	9.12	0.665–13.3
*γ*_0_	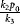	Scaled protein switch *p*_0_	1.94	0.542–2.58
*γ*_1_	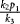	Scaled protein switch *p*_1_	1.91	0.516–3.18
*γ*_2_	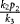	Scaled protein switch *p*_2_	2.23	0.647–3.11
*Ω*_1_	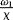	Scaled oxygen switch *ω*_1_	0.0047	0.0025–0.01
*Ω*_2_	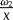	Scaled oxygen switch *ω*_2_	0.103	0.0433–0.195

## Appendix A

The non-dimensional standard model is

where  is the non-dimensional input function and Table II presents standard values for the non-dimensional parameters.

## Nomenclature

### Abbreviations

*a*: maximum rate of uptake of external sulfur (μM h^−1^)
*A*_1_: dimensionless ratio of sulfur uptake rates
*A*_2_: dimensionless ratio of sulfur uptake rates
ATP: adenosine triphosphate
*b*_*i*_: rate constants for sulfur uptake (*i* = 1, μM)
*b*_w_: width of the bio-reactor (cm)
*B*: dimensionless ratio of S uptake rates
*D*_C_: dimensionless absorption measure
*e*: endogenous substrate (model variable)
*E*_L_: fraction of electrons from PSII-dependent path
input of external sulfur function (Equation (4))
*g*: gradient of the smoothed Heaviside switch function
*G*: dimensionless scale factor in *α*(*s*)
*h*: hydrogen gas (model variable) (mL L^−1^ culture)
*H*_“name”_: heaviside function to model process, “name”
*I*_0_: dimensionless light intensity at the source
*I*_sat_: dimensionless saturation level of light
function for total light intensity averaged over width
*k*_1_: rate constant for PSII repair (h^−1^)
*k*_2_: rate constant for protein breakdown (h^−1^)
*k*_3_: rate constant for protein production (μM h^−1^)
*k*_4_: rate constant for hydrogen production (mL h^−1^)
*k*_5_: rate constant for oxygen consumption by respiration (h^−1^)
*k*_6_: rate constant for oxygen production from PSII (μM h^−1^)
*k*_chl_: measure of absorbance of the cells (cm^2^)
*K*_3_: dimensionless measure of rate of protein production
*K*_2_: dimensionless protein breakdown rate
*K*_5_: dimensionless respiration rate
*K*_6_: dimensionless photosynthesis rate
*L*(*Λ*): dimensionless usable light intensity (Equation (2))
*L*_*e*1_: value of the usable light function in Kosourov et al. (2002) (μmol m^−2^ s^−1^)
*n*: cell concentration (cells/mL)
*n*_0_: uniform cell concentration throughout the layer (cells/mL)
NADP^+^: nicotinamide adenine dinucleotide phosphate
NADPH: reduced form of NADP^+^
*p*: protein (model variable) (μM)
*p*_0_: protein level when growth is zero (μM)
*p*_1_: protein below which maximum decay occurs (μM)
*p*_2_: protein required for maximum growth (μM)
*p*_*h*_: normalization of PSII-independent electron pathway (μM)
*p*_*r*_: basic protein needed for cell survival (μM)
*P*_G_: dimensionless protein gradient
*P*_H_: dimensionless reciprocal of *p*_*h*_
*P*_R_: dimensionless protein required for survival
PSI/PSII: photosystem I/II
*r*_decay_: maximum rate for cell decay (h^−1^)
*r*_exp_: maximum growth rate (h^−1^)
*R*_D_: dimensionless decay rate
*G*_*i*_: dimensionless growth rate
*s*: internal sulfur (model variable) (μM)
*s*_n_: normal level of sulfur in a cell (μM)
*s*_1_: sulfur level above which Calvin cycle is active (μM)
*s*_h_: normalization of PSII-dependent electron pathway (μM)
*S*: external sulfur (model variable) (μM)
*S*_0_: initial external sulfur (μM)
*S*_1_: ratio of sulfur required for Calvin cycle compared to normal sulfur concentration
*S*_H_: dimensionless reciprocal of *s*_h_
*t*: time
*T*: time at which total hydrogen yield *h* is output
*T*_H_: start time of hydrogen production
*T*_i_: scaled time period over which initial hydrogen production rate is measured
oxygen mass transfer coefficient
*V*_cell_: volume of a single cell (mL)
*V*_container_: volume of the container (mL)
*V*_L_: dimensionless oxygen mass transfer
*α*(*s*): function for the maximum uptake rate of external sulfur (Equation (3))
*β*(*s*): function for the half saturation value of *S* uptake (Equation (3))
*β*: average moles of sulfur in one mole of protein
*γ*_0_: dimensionless protein switch *p*_0_
*γ*_1_: dimensionless protein switch *p*_1_
*γ*_2_: dimensionless protein switch *p*_2_
*Λ*: cell volume fraction (model variable)
cell volume fraction when hydrogen production begins
*χ*: oxygen saturation in water (μM)
*ω*: oxygen (model variable) (μM)
*ω*_1_: oxygen level required for full respiration (μM)
*ω*_2_: oxygen level required to inhibit H_2_ production (μM)
*ω*_p_: oxygen level below which protein breakdown occurs (μM)
*Ω*_1_: dimensionless oxygen switch *ω*_1_
*Ω*_2_: dimensionless oxygen switch *ω*_2_
